# Decoding RAS isoform and codon-specific signalling

**DOI:** 10.1042/BST20140057

**Published:** 2014-08-11

**Authors:** Anna U. Newlaczyl, Fiona E. Hood, Judy M. Coulson, Ian A. Prior

**Affiliations:** * Division of Cellular and Molecular Physiology, Institute of Translational Medicine, University of Liverpool, Liverpool L69 3BX, U.K.

**Keywords:** cancer, GTPase, HRAS, KRAS, NRAS

## Abstract

RAS proteins are key signalling hubs that are oncogenically mutated in 30% of all cancer cases. Three genes encode almost identical isoforms that are ubiquitously expressed, but are not functionally redundant. The network responses associated with each isoform and individual oncogenic mutations remain to be fully characterized. In the present article, we review recent data defining the differences between the RAS isoforms and their most commonly mutated codons and discuss the underlying mechanisms.

## Introduction

RAS proteins are membrane-bound small GTPases that act as molecular switches downstream of cell-surface receptors. Upon GTP binding, RAS changes conformation, revealing an effector-binding site that can interact with proteins harbouring a RAS-binding domain [1]. Relocalization of effectors to the membrane promotes interactions with their regulators, substrates and other effectors inducing a signalling response. These include activation of the RAF/MAPK (mitogen-activated protein kinase) pathway that promotes cell proliferation and the PI3K (phosphoinositide 3-kinase)/Akt pathway that promotes cell survival [2].

In the human genome, three genes encode HRAS, KRAS and NRAS. Alternative splicing of HRAS and KRAS generates protein variants with altered C-termini [3]. The N-terminal half of the RAS isoforms share complete amino acid identity; this includes the effector binding domain and the majority of residues responsible for co-ordinating GDP/GTP binding, GTPase activity and regulators of RAS activity. The major area of protein sequence divergence between the isoforms is found in the final 26 amino acids. This region is post-translationally modified to enable membrane binding and correct localization of each of the RAS isoforms [4].

Despite their similarity, RAS proteins are not functionally redundant. Furthermore, the oncogenic mutations that make RAS constitutively active may not all be equivalent in their functional consequences. We discuss the evidence for this together with the potential mechanisms underlying these phenomena.

## Oncogenic RAS

**Figure 1:**
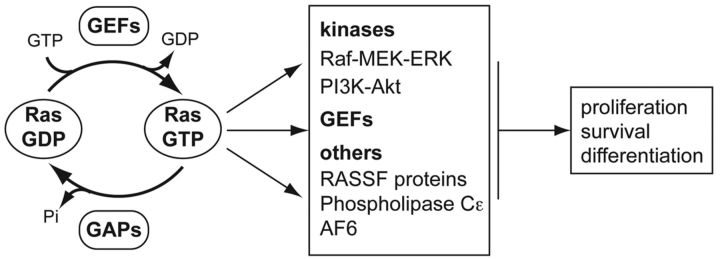
RAS activation and signalling RAS cycling between the inactive GDP-bound and the active GTP-bound conformations is controlled by GEFs and GAPs. GTP-bound RAS can interact with more than 20 effectors that control many cellular functions. AF6, ALL-1-fusion partner in chromosome 6; ERK, extracellular-signal-regulated kinase; MEK, MAPK/ERK kinase; RASSF, RAS-association domain family.

RAS proteins cycle between the inactive GDP-bound and the active GTP-bound conformations (Figure 1). This cycle is controlled by regulatory GEFs (guanine-nucleotide-exchange factors) and GAPs (GTPase-activating proteins) [5]. Mutations of RAS at codons 12, 13 or 61 render the protein insensitive to GAP function and/or impair GTPase activity, resulting in a constitutively active protein that promotes oncogenesis [6]. Importantly, all three RAS isoforms share complete sequence identity at these sites and the mutations are assumed to have equivalent effects on the activity of each isoform [4]. Despite this, KRAS is the most frequently mutated isoform and mutation of each RAS protein exhibits coupling with a specific subset of cancers [7,8]. For example, KRAS is strongly associated with pancreatic, colorectal and lung cancers, whereas NRAS is the isoform most frequently mutated in haemopoietic tumours. HRAS mutations are rarely detected in tumours.

The reason for the bias in RAS mutations associated with individual cancers is unclear, although several models have been proposed. One simple possibility is that isoform-specificity could mean that a more oncogenic network response is generated when KRAS is rendered constitutively active by mutation compared with the other RAS isoforms. This is supported by mouse models harbouring G12D mutated KRAS that exhibited widespread colonic hyperplasia and neoplasia. In contrast, NRAS G12D stimulated cell survival pathways [9]. Further support for the oncogenic potency of the KRAS signalling network comes from comparative studies utilizing ectopic expression of G12V mutated RAS isoforms. This revealed that KRAS promotes endodermal stem cell expansion, whereas NRAS and HRAS do not [6,10]. This is significant because many cancers of endodermal origin such as pancreatic, lung and colorectal cancer are strongly associated with KRAS mutations. This suggests a model where the KRAS coupling to specific cancers is due to its role in expanding cancer progenitor cells in these tissues.

**Figure 2:**
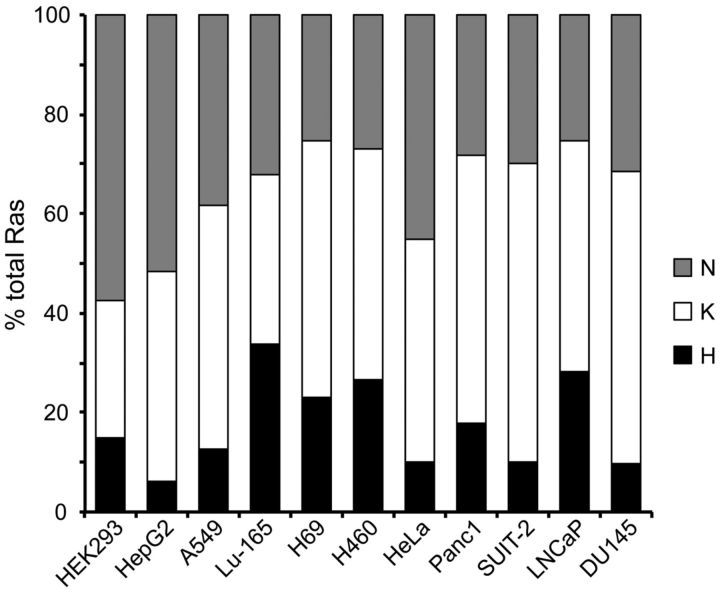
Relative RAS isoform protein abundance in a panel of cancer cell lines KRAS is the most abundant isoform in the majority of cell lines, whereas HRAS typically represents ≤20% of total RAS in most cell lines. Data taken from [43].

Recently, an alternative model for KRAS mutation predominance in cancer was suggested on the basis of the likely relative protein expression levels of each isoform. Analysis of the DNA sequence of *KRAS* revealed a strong bias for rare codon usage resulting in significantly reduced protein translation compared with a codon-optimized *KRAS* sequence [11]. Overexpression of oncogenically mutated *RAS* is known to induce senescence rather than proliferation [12]. The authors speculated that the rare codon usage in *KRAS* leads to low endogenous protein levels, meaning that oncogenically activated *KRAS* would not be expressed highly enough to induce the senescent phenotype. Absolute quantification of RAS isoform protein abundance in normal tissues has not been performed to date; however, comparative analysis of RAS isoform protein levels in a panel of cancer cell lines revealed that KRAS is typically the most abundant isoform (Figure 2). Therefore whether the preponderance of KRAS mutations in cancer is linked to relative abundance of the isoforms remains an open question.

Finally, whereas RAS isoforms are identical at the amino acid level in the region containing the mutated codons, their DNA sequences differ. Differences in adduct repair rates between the isoforms may explain the abundance of KRAS mutations. Evidence for this comes from Tang and co-workers who found that tobacco smoke mutagen adducts at codon 12 of *KRAS* were repaired less efficiently than those in *HRAS* or *NRAS* [13,14]. The mechanistic basis for this may be due to local sequence-dependent differences in DNA curvature that are recognized by the DNA repair enzymes.

**Figure 3:**
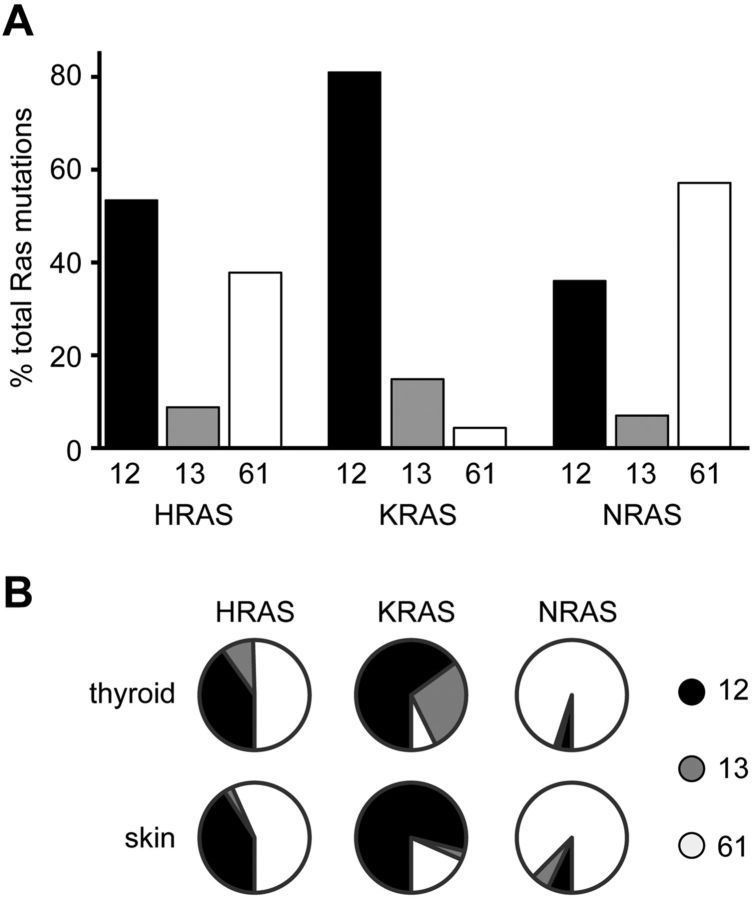
RAS isoform codon mutation bias in human cancers (**A**) A survey of RAS mutations across all cancers reveals that *KRAS* favours mutation at codon 12, whereas *NRAS* is typically mutated at codon 61. (**B**) Analysis of individual tumour types with ≥20 mutations of each isoform reveals similar codon biases. Data from Sanger COSMIC (Catalog of Somatic Mutations in Cancer) version 52 dataset and collated in [8].

Mutations at codons 12, 13 and 61 account for over 99% of all RAS mutants detected in cancer [8]. An intriguing feature is the preference of each isoform for mutation at different codons, with *NRAS* being typically mutated at codon 61 whereas 80% of *KRAS* mutations are at codon 12 (Figure 3A). Single base substitutions can result in conversion into six other amino acids at codons 12, 13 and 61. Despite this, over 60% of the total mutations observed for each isoform are accounted for by only three of the 18 possible codon variants [8]. RAS isoform-specific bias is also evident in which mutations are typically seen at each codon. For example, G12V mutations are ten times more abundant than G12D mutations in HRAS cancer samples, whereas for KRAS and NRAS G12D mutations predominate [8].

Some of the mutational heterogeneity will be due to tissue-specific exposure to different cocktails of mutagens. For example, the G12C mutation observed in KRAS in lung cancers is associated with exposure to tobacco smoke mutagens [15]. However, this does not explain why there is still codon mutation bias between isoforms within the same cancer. Thyroid and skin cancers are the only two cancer types where substantial numbers of mutations of all three isoforms are observed. In both cases, the bias towards codon 61 mutations for *NRAS* and codon 12 mutations for *KRAS* is clear (Figure 3B).

Given that mutations at codons 12, 13 and 61 are all activating in all of the isoforms, does it matter which codon is mutated or which substitution has occurred? Cell transformation is used as a read-out for oncogenic RAS function, and early comparative studies revealed a spectrum of transforming capabilities among *HRAS* codon 12 and codon 61 mutants [16,17]. *In vitro* experiments revealed that cells expressing *KRAS* codon 12 mutations were more resistant to apoptosis than codon 13 mutant cells [18]. More recently, direct clinical support for codon-specific signalling was observed in colorectal cancer. Patients with *KRAS* codon 13 mutations have a worse prognosis than those with *KRAS* mutated at codon 12 [19,20]. A first-line therapy for patients with advanced colorectal cancer is the anti-EGFR (epidermal growth factor receptor) drug cetuximab; however, this is was thought to be ineffective for patients with KRAS mutations. For this reason, routine screening of colorectal cancer patients for KRAS mutations is now carried out. However, meta-analysis of cetuximab resistance data found that patients with KRAS G13D mutations showed significant improvements in survival [20]. Finally, different codon mutations are differentially sensitive to allosteric regulation of RAS that normally stimulates intrinsic GTPase activity. Codon 61 but not codon 12 is critical for stabilizing this allosteric switch required for promoting GTP hydrolysis [21]. However, codon 61 mutants are switched into the anti-catalytic conformation when bound to RAF, this means that these mutants are particularly potent activators of the RAF/MAPK pathway compared with the other codon mutants [22].

Therefore the functional consequences of different RAS mutations are not necessarily equivalent. This represents an important emerging concept that is likely to inform the design of experiments, screens and trials in the coming years.

## Isoform-specific RAS signalling

Differential RAS isoform mutation frequencies and coupling to individual cancers suggests isoform-specific function. Other *in vivo* support is provided by studies of mouse development where KRAS knockout is embryonic lethal [23]. In contrast, double knockout of HRAS and NRAS produced viable offspring with no obvious negative phenotypic consequences [24]. Comparative *in vitro* studies involving ectopic overexpression of each of the isoforms revealed preferential coupling of KRAS with the RAS/MAPK pathway and HRAS and NRAS with PI3K/Akt activation [25,26].

The mechanistic basis for isoform-specific signalling is thought to lie, at least in part, in the different trafficking and overlapping, but distinct, subcellular localizations of each isoform [27]. This is governed by the 25/26-amino-acid C-terminal hypervariable region that is post-translationally modified to allow membrane binding [28]. All RAS isoforms contain a C-terminal CAAX motif that is farnesylated before proteolysis to remove the -AAX residues and carboxymethylation [29]. The weak membrane affinity is supplemented by either palmitoylation in the case of HRAS, NRAS and the KRAS4A splice variant or a stretch of basic lysine residues in KRAS4B [30]. Palmitoylation specifies trafficking via the Golgi to the plasma membrane and differential localization within cell surface nanoclusters [31–33]. This modification is reversible, and depalmitoylation allows a dynamic flux between the cell surface and the Golgi complex [34,35]. Electrostatic interactions direct reversible KRAS4B localization with the plasma membrane [31,32]. In contrast with the palmitoylated RAS isoforms, almost no endomembranous localization is observed for KRAS4B [4]. Although the plasma membrane is the main site of action of RAS, subcellular organelles have been shown to be competent to support RAS function [36–38]. Differences in localization between the RAS isoforms are thought to bring them into contact with different concentrations of regulators and effectors to generate overlapping, but distinctive, outputs [27,39].

A second mechanism for generating isoform-specific signalling is via differences in the presentation of the effector-binding domain. Upon GTP binding, the G-domain (residues 1–166) of RAS isoforms change their orientation with respect to the membrane [40]. The extent of this reorientation varies between the isoforms and binding of the key RAS effectors PI3K and RAF is sensitive to these changes. RAF binding is favoured by the KRAS orientation, whereas PI3K binding favours that adopted by HRAS [41].

Finally, relative expression differences of the isoforms will influence competition for regulator and effector binding and therefore signalling network responses. An example of this can be seen in studies of mouse development. Whereas *KRAS* knockout resulted in embryonic lethality [23], a subsequent study inserted *HRAS* into the endogenous *KRAS* locus, resulting in normal embryonic development, but induced dilated cardiomyopathy during adulthood [42]. This suggests that HRAS can functionally replace KRAS during early development, but only when expressed under the control of KRAS-regulatory promoter regions.

This illustrates the importance of studying isoform-specific function in an endogenous context, since a subset of isoform-specific differences may result from the disparity in their expression. Future work is likely to rely less on ectopic overexpression and more on isogenic cells harbouring endogenous wild-type and mutant *RAS* alleles, panels of cancer cell lines and mouse models.

## Conclusions

RAS proteins exhibit a spectrum of isoform-, codon-, point-mutation- and context-specific network responses. These remain poorly understood, which precludes simple prediction of the likely consequences of any given mutation. Wherever possible, it is important to study RAS function in an endogenous context to avoid the perturbing influence of overexpression on the signalling networks. With the recent emergence of new cellular tools, xenopatient and genetically engineered mouse models and more sophisticated systems biology approaches to studying RAS function, there are likely to be significant improvements in our understanding of RAS biology in the near future.

## Abbreviations

GAP: GTPase-activating protein
GEF: guanine-nucleotide-exchange factor
MAPK: mitogen-activated protein kinase
PI3K: phosphoinositide 3-kinase
